# Effect of Sweet Potato Vine on the Onset of Puberty and Follicle Development in Chinese Meishan Gilts

**DOI:** 10.3390/ani9060297

**Published:** 2019-05-30

**Authors:** Pan Zhang, Meng Cao, Jian Li, Yan Lin, Zhengfeng Fang, Lianqiang Che, Bin Feng, Yong Zhuo, Jianping Wang, De Wu, Shengyu Xu

**Affiliations:** Key Laboratory of Animal Disease-Resistant Nutrition, Ministry of Education; Key Laboratory of Animal Disease-Resistant Nutrition and Feed, Ministry of Agriculture and Rural Affairs; Key Laboratory of Animal Disease-resistant Nutrition, Sichuan Province; Animal Nutrition Institute, Sichuan Agricultural University, Chengdu 611130, China; zhangpan5335@sina.cn (P.Z.); caomeng1111@126.com (M.C.); lijian522@hotmail.com (J.L.); able588@163.com (Y.L.); fangzhengfeng@hotmail.com (Z.F.); clianqiang@hotmail.com (L.C.); fengb123d@163.com (B.F.); zhuoyong@sicau.edu.cn (Y.Z.); wangjianping1983@hotmail.com (J.W.)

**Keywords:** Chinese Meishan gilts, sweet potato vine, puberty onset, follicular development, granulosa cells

## Abstract

**Simple Summary:**

Sweet potato vine, as a by-product of sweet potato, has been previously used for various purposes in southern China. The aim of this study was to investigate the effects of sweet potato vine on the onset of puberty and follicular development in the ovaries of Chinese Meishan gilts. Our results suggested that sweet potato vine supplementation delayed puberty onset and improved the follicular development, which was associated with enhanced reproductive performance in Chinese Meishan gilts.

**Abstract:**

This study was conducted to evaluate the effects of sweet potato vine on the onset of puberty and the follicular development in the ovaries of Chinese Meishan gilts. A total of 20 Meishan gilts (initial body weight at 30 ± 0.18 kg) were randomly fed a control (CON) or sweet potato vine (SPV) supplemented diet until 19 days following the third estrous. Sweet potato vine was instead of part of basal diet with the same amount of energy and protein in the sweet potato vine group. The results indicate that gilts fed with sweet potato vine reached puberty 9.4 days later. The development of ovaries was enhanced by sweet potato vine supplementation, characterized by an increase (*p* < 0.05) in the relative weight of the ovaries and the number of large follicles (>5 mm). Sweet potato vine supplementation increased (*p* < 0.05) the total superoxide dismutase (T-SOD) and reduced (*p* < 0.05) the concentration of malondialdehyde (MDA) in the serum of the gilts. Also, the expression of superoxide dismutase-1 (SOD1) and luteinizing hormone receptor (LHR) mRNA in the granulosa cells from the large follicle (>5 mm) of gilts in the SPV group were increased (*p* < 0.05) as compared with the CON group. These results indicate that gilts fed with sweet potato vine exhibited delayed puberty as well as improved follicular development, which may contribute to the reproductive performance of Chinese Meishan gilts.

## 1. Introduction

Sweet potato is a type of root crop commonly cultivated in southern China. Sweet potato vine (SPV) consisting of leaf, stem, and stalk is the by-product of its root and also a form of green forage. It’s considered an ideal feedstuff for pigs in the south of China due to its lower price and easy access, and SPV has been used as a protein and fiber source in pig diets, especially local Chinese pig breeds. Previous studies have shown that the intake of sweet potato leaves stimulates swine growth [1], and high protein sweet-potato roots were used as pig feed and the performance of pigs was better than those fed corn meal [2]. However, the proportion of sweet potato vines in pig diets should not be excessively high to avoid adverse effects on nutrient digestibility [3]. This can be due to the increase in fiber content of the diets [4] and the effect of the trypsin inhibitors in SPV. The Chinese Meishan (MS) pig is famous for its high reproductive performance, and thus the factors responsible for this have been extensively studied [5]. Nutritional factors are key predictors of puberty timing other than heredity and environmental factors [6]. For gilts, the appropriate onset of puberty and appropriate follicular development has been suggested as indicators of lifetime reproductive performance [7,8]. The onset of puberty is the process of physiological change by which an organism gradually reaches sexual maturity to reproduce offspring, mainly reflected in follicular development and oocyte maturation. In mammals, granulosa cells (GCs) are a type of granular-like cell formed by the differentiation of the follicular cells on the inner wall of the follicular cavity, which support the development of primordial follicles into primary follicles, secondary follicles, and antral follicles [9,10,11], and are also involved in the recruitment, selection, atresia, ovulation, and luteolysis of follicles [9,10,12]. Moreover, GCs are one kind of important endocrine cell in the ovary. GCs express luteinizing hormone (LH) receptor and follicle stimulating hormone (FSH). They are the main cells in the ovary responsive to LH and FSH signals.

It is well known that the MS pig originates in China’s Yangtze River basin and relies on green forage [13]. Green forage seems to have specific reproductive actions in MS pigs. Studies have demonstrated that sows fed with green forage (fresh alfalfa or fresh ryegrass) exhibit improved reproductive performance [14,15]. However, there are no reports on whether sweet potato vine affects the onset of puberty in MS gilts.

Therefore, the objectives of this study were to investigate the effects of sweet potato vine on the onset of puberty and ovarian follicle development in MS gilts. It was hypothesized that sweet potato vine offered to MS gilts would alter the follicle development when sweet potato vine instead of basal diet with the same amount of energy and protein.

## 2. Materials and Methods

The experimental procedures for this study were approved by the Animal Experimental Committee of Sichuan Agricultural University (Ethic Approval Code: SCAUAC201408-10).

### 2.1. Animals and Experimental Design

Twenty prepubertal MS gilts, with an initial body weight at 30 ± 0.18 kg (109.00 ± 2.10 day) were used in this study. Gilts were randomly allocated to one of the two groups with 10 animals for each group: control (CON) and sweet potato vine (SPV) groups. The control basal diets were formulated to meet or exceed Chinese Feeding Standard of Swine (2004) (Table 1). Sweet potato vine (Table 1) was part of the basal diet with the same amount of energy and protein in the SPV group. The feeding strategy for the Chinese Meishan gilts is shown in Table 2. After sweet potato vine was chopped, it was mixed with the basal diet and fed to the gilts in the SPV group. All the gilts were fed twice daily (08:00 and 14:00) and water was available ad libitum from a nipple drinker. All the gilts from the two groups were housed individually in a stall (3 × 2 m).

### 2.2. Data Record and Sample Collection

Throughout the study, gilts were exposed to a rotation of mature MS boars twice a day (08:30 and 16:30). Meanwhile, estrous detection was carefully conducted by an experienced stockperson based on behavioral and vulvar characteristics. The day of the gilt expressing initial estrous behavior (indicating a standing reflex in response to the manual application of pressure to the gilt’s back (back pressure test)) was recorded as the age of puberty onset. Then, each estrous cycle as well as the duration of each estrus cycle were recorded. The day after detecting the standing reflex on the third estrus cycle of the gilt was designated as Day 1. On the morning of Day 19, the live weight of all gilts (*n* = 10/each group) was recorded, and then 10 mL of blood sample was collected by acute jugular puncture prior to slaughter. All blood samples were centrifuged immediately after collection (2400× g for 15 min at 4 °C) and the serum samples were stored at −20 °C until analysis.

The gilts from each group were slaughtered by an intracardial injection of sodium pentobarbital (50 mg/kg body weight) and bled by exsanguination after blood sampling. After the gilt was slaughtered, the reproductive tract was dissected and the right and left ovaries were separated from the uterine horns and weighed. The uterus was subsequently trimmed of mesentery and weighed. All the antral follicles greater than 1 mm in diameter were individually measured and recorded within different size categories (small: <3 mm in diameter, medium: 3 to 5 mm in diameter and large: >5 mm in diameter). Meanwhile, the number of corpora lutea was recorded. Then, the follicular content was aspirated from all the follicles with a diameter > 5 mm using a 10-mL syringe equipped with an 18-gauge needle. Following aspiration, the follicular contents were centrifuged for 5 min at 3000 rpm to obtain the clear follicular fluid and the cell pellet. The supernatant (follicular fluid) was collected and stored at −20 °C until reproductive hormone analysis. The cumulus-oocyte complexes (COCs) were taken away from the cell pellet using a dissecting microscope after dissociation with phosphate-buffered saline. Then they were centrifuged for 5 min at 3000 rpm to obtain the GCs and stored at −80 °C until analysis.

### 2.3. Serum and Follicular Fluid Analyses

The concentrations of insulin-like growth factors-1 (IGF-1) and estradiol (E_2_) in serum and follicular fluid were measured using non-competitive enzyme-labeled immunosorbent assays (ELISA) kits (Nanjing Jiancheng Bioengineering Institute, Nanjing, China). The minimum detectable concentrations of IGF-1 and E_2_ using the kits were estimated to be 0.01 ng/mL and 0.01 ng/mL, respectively. All the hormone assays were performed in 96-well plates using an enzyme-labeled meter (Thermo Electron Corporation, Waltham, MA, USA), according to the manufacturer’s protocol. Intra- and inter-assay CVs of IGF-I and E_2_ were 5.6% and 6.7%, 6.8 and 6.4%, respectively.

### 2.4. Oxidative Stress Biomarkers

Serum antioxidant parameters were analyzed by commercially available kits (Nanjing Jiancheng Bioengineering Institute, Nanjing, China), according to the manufacturer’s instructions. All the samples were measured in duplicate. After thawing serum samples in ice-cold buffers, the activities of total antioxidative capability (T-AOC), glutathione peroxidase (GPX), total superoxide dismutase (T-SOD), malondialdehyde (MDA), and Vitamin E (VE) level were determined using the colorimetric methods with Beckman DU-800 UV-visible spectrophotometer (Beckman Coulter Inc., Fullerton, CA, USA). Briefly, MDA content were measured based on the thiobarbituric acid (TBA) method. The activities of T-AOC, GPx, T-SOD, and VE were assayed as described by Xu et al. [18].

### 2.5. Total RNA Extraction and Real-Time RT-PCR

Total RNA was extracted with TRIzol reagent (Invitrogen, Carlsbad, CA, USA) from frozen GCs. The RNA was treated by DNase-I (TaKaRa Biotechnology Co., Ltd., Dalian, China) for removing trace quantity DNA and quantified spectrophotometrically. cDNA was synthesized with random primers (Invitrogen, Carlsbad, CA, USA). Real-time PCR was used to quantify glutathione peroxidase (*GPx*), superoxide dismutase-1 (*SOD1*), superoxide dismutase-2 (*SOD2*), follicle-stimulating hormone receptor (*FSHR*), luteinizing hormone receptor (*LHR*), and estrogen receptor-α (*ER*α) mRNA expression levels using the Sybr Green Kit (Qiagen, Valencia, CA, USA). Primers were listed in Table 3. Amplification was carried out with 12.5 µL final reaction volume containing 5 µL of Sybr green master mix, 0.5 µL of each primer (final concentration 0.25 µM), 6 µL of diethyl pyrocarbonate (DEPC)-water, and 0.5 µL of sample cDNA. All real-time PCRs were carried out in triplicate on a DNA Engine thermal cycler (PTC-0200, Chromo4 Real-Time detector, Bio-Rad, Hercules, CA, USA) using thermal cycling conditions (denaturation for 10 min at 95 °C, amplification for 45 cycles with denaturation at 95 °C for 20 s, annealing at 60 °C for 10 s, extension at 72 °C for 9 s followed by fluorescence acquisition at 60 °C for 5 s). To get rid of the potential contamination, one reaction with the cDNA replaced by water was applied. Product sizes were verified by agarose gel electrophoresis and all products were sequenced to confirm identity. The housekeeping gene β-actin was amplified for each sample to verify the presence of cDNA and as an internal control to calculate the relative level of target gene expression using the 2^−∆∆*C*t^ method [19].

### 2.6. Statistical Analysis

All data are expressed as means ± standard error of the mean (SEM). Data were analyzed using SPSS 21.0 statistical software program (IBM SPSS Company, Chicago, IL, USA). An independent t-test was used to detect differences between the two diet groups and relative differences in the target gene of GCs were determined by the 2^−∆∆*C*t^ method. Differences were considered significant at *p* ≤ 0.05, whereas 0.05 < *p* < 0.10 was considered as a tendency.

## 3. Results

### 3.1. Effect of Sweet Potato Vine on the Estrous and Follicular Development in Meishan Gilt

The Meishan gilts fed with sweet potato vine reached estrous 9.4 days later than those in the CON group (*p* = 0.08, Table 4) and tended to have higher body weight at the first estrus (*p* = 0.09), but there was no difference in the estrous cycles as well as in the duration of the first and second estrous cycles between the two groups.

As shown in Table 5, the ovarian and uterine weight were greater (*p* < 0.05) in the SPV group than those in the CON group. The relative weights of ovaries from gilts fed with sweet potato vine were heavier (*p* = 0.05) than those in the CON group, but there was no significant difference in the relative weights of the uterus between the two groups. The results in the follicular development assessment indicated that the number of large follicles (>5mm) was greater (*p* < 0.05) in the SPV group of gilts than in the CON group. Compared with CON group, the SPV group had a trend of an increase (*p* = 0.06) in the number of corpora lutea. However, there was no difference in the number of medium follicles and small follicles between the two groups.

### 3.2. Effect of Sweet Potato Vine on the Concentration of Hormone in the Serum and Follicular Fluid

The data on concentrations of hormones in Meishan gilts fed with sweet potato vine reveal higher concentrations of E_2_ and IGF-1 (70.67 ± 2.35 vs. 63.70 ± 1.30 ng/mL, *p* = 0.02; 210.00 ± 12.30 vs. 177.00 ± 9.10 ng/mL, *p* = 0.05, respectively) in the follicular fluid compared to the CON group. However, there were no treatment differences in the concentrations of E_2_ and IGF-1 (95.00 ± 8.60 vs. 87.00 ± 14.40 pg/mL; 215.20 ± 21.70 vs. 186.60 ± 12.40 ng/mL, *p* > 0.05, respectively) in the serum.

### 3.3. Effect of Sweet Potato Vine on the Oxidative Stress Status in Meishan Gilts

The Meishan gilts in the SPV group had higher (121.20 ± 1.50 vs. 116.10 ± 1.60 U/mL, *p* < 0.05) serum concentrations of T-SOD and lower (1.40 ± 0.30 vs. 2.60 ± 0.40 nmol/mL, *p* < 0.05) serum concentrations of MDA than those in the CON group. However, there were no significant treatment differences in the serum concentrations of T-AOC, GPx, and VE (1.10 ± 0.20 vs. 0.90 ± 0.20 U/mL; 1273.20 ± 39.90 vs. 1218.30 ± 49.90 U/mL; 3.30 ± 0.40 vs. 2.90 ± 0.40 μg/mL; *p* > 0.05, respectively).

### 3.4. Effect of Sweet Potato Vine on Antioxidant Stress and Hormone Receptor Related Gene Expression

**Figure 1:**
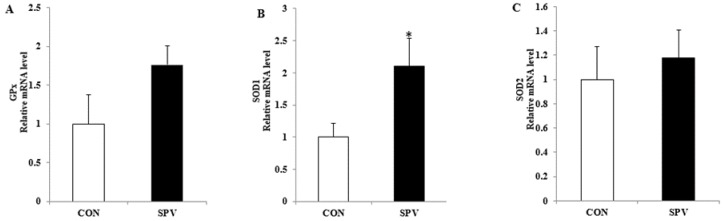
Effect of sweet potato vine supplementation on antioxidant stress relative genes’ mRNA expression in the granulosa cells (*n* = 10). Data are presented as mean ± SEM. CON, gilts feed the basal diet; SPV, gilts fed basal diet with sweet potato vine. *GPx*, glutathione peroxidase; *SOD1*, superoxide dismutase-1; *SOD2*, superoxide dismutase-2. * *p* ≤ 0.05.

We also evaluated mRNA expression levels of antioxidant enzymes in GCs (Figure 1). Compared with control diets, Meishan gilt fed sweet potato vine diets had increased (*p* < 0.05) gene expression level of *SOD1* in the GCs derived from large follicles (>5 mm; Figure 1B). However, there was no treatment difference in *GPx* and *SOD2* gene expression.

**Figure 2:**
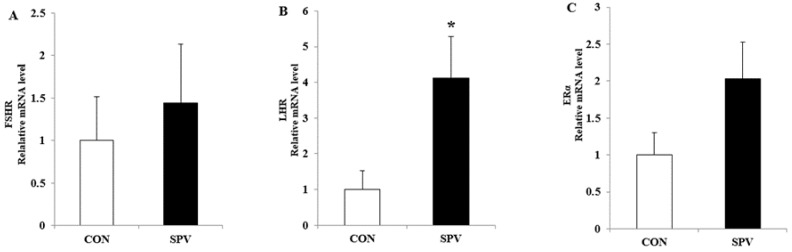
Effect of sweet potato vine supplementation on hormone receptor relative genes’ mRNA expression in the granulosa cells (*n* = 10). Data are presented as mean ± SEM. CON, gilts feed the basal diet; SPV, gilts fed basal diet with sweet potato vine. *FSHR*, follicle-stimulating hormone receptor; *LHR*, luteinizing hormone receptor; *ERα*, estrogen receptor-α. * *p* ≤ 0.05.

The expression of *LHR* mRNA in GCs from the large follicle (>5 mm) of gilts in the SPV group was increased (*p* < 0.05, Figure 2) as compared with the CON group, whereas the expression of *FSHR* and *ERα* mRNA in the GCs were not significantly affected.

## 4. Discussion

In addition to key environmental factors which affect the timing of puberty, diet is also closely related to the reproductive performance and longevity of gilts. Sweet potato vine, which is rich in vitamins and fiber, seems to exhibit specific actions in MS pigs and modulate reproductive performance. In the present study, the gilts in the SPV group reached puberty 9.4 days later than those in the CON group, indicating that replacing part of basal diet with sweet potato vine delayed the onset of puberty in MS gilts. Although some studies suggest that gilts with delayed puberty had poorer subsequent reproductive performance [20], Tummaruk et al. (2001) [21] found that if the age at first mating of the gilts was delayed for 10 days, there would be a 0.1 piglet increase in the first litter. It was also found that gilts mated at a younger age are culled later in life than gilts mated at an older age [8]. In this study, the slaughtered age of gilts in the SPV group, which is presumed to be the time for mating, was delayed by 9.7 days (Table 4), there was no difference in the duration of the first and second estrous cycles between the two groups, so the difference between treatments for the age at slaughter and weight at slaughter are closely related to the age at puberty. The increase in the slaughter age of MS gilts in the SPV group was attributed to the delayed estrus. The delayed onset of puberty caused by sweet potato vine may contribute to the reproductive performance in primiparous MS sows.

Prior studies indicate that the gilts which received green forage (alfalfa) diet have increased ovulation rates and produce more live pigs per litter [22]. There is additional evidence of effects of altered nutritional regimens prior to mating on follicular development in pigs. In the present study, improved ovarian development of gilts in the SPV group was characterized by an increase in the relative weight of ovary as well as in the number of large follicles and corpus lutea. The ovary is one of the most important organs for reproduction [23] and a well-developed ovary could provide an excellent environment for development of follicles and oocytes. Although all the stages of follicles play an important role in ovulation, the number of large follicles could reflect the minimum ovulatory population. A larger diameter of the follicles may lead to higher quality oocytes [24]. For gilts, an assumption of the largest follicles in the late follicular phase representing the presumptive ovulatory population was considered [25]. Compared with smaller follicles, larger follicles provided a better environment, including sufficient ribonucleoside, amino acids, and phospholipids to support the maturation of the oocyte [9]. Thus, in the current study, the gilts in the SPV group had better follicular development before mating, resulting in higher oocyte quality. Follicle development and oocyte maturation are a complex process of endocrine, autocrine, and paracrine effects coordinated by multiple hormones and growth factors. Metabolic hormones such as insulin, IGF-I, and leptin are likely to be mediators of nutritional status and reproductive performance [26]. In this study, the concentration of IGF-I in the follicular fluid of gilts in SPV group was increased. Previous studies have proposed that pig oocytes matured *in vitro* in culture medium supplemented with IGF-I have a higher proportion of oocytes able to mature. Also, previous studies have identified a close relationship between the concentration of IGF-I in the follicular fluid, follicle size [27] and the concentration of IGF-I in the follicular fluid was effective in stimulating the proliferation in the GCs *in vitro* [28]. Furthermore, IGF-I enhance the production of FSH-induced aromatase and LH receptor expression in the GCs [29] and increased the responsiveness to FSH in a granulosa cell culture [30]. In this study, the gilts fed with sweet potato vine exhibited higher expression levels of *LHR* gene in GCs from the large follicles (> 5 mm). The *LHR* gene is expressed in the GCs for the entire follicular phase. LHR mediates LH, which works with FSH to promote follicular endometrial cells to produce androgen, and is a precursor to estrogen synthesis by the granular cells [31]. In agreement with this last finding, the present experiment indicated that E_2_ was increased in the follicular fluid in the sweet potato vine supplementation group compared with the control group. Porcine follicles become dominant when they reach 3 mm in diameter, and then switch from FSH-dependent to LH-dependent [32]. The *LHR* expression was the greatest in the GCs from preovulatory follicles just prior to the induction of ovulation [33]. Thus, in the present study the increased expression of *LHR* mRNA in GCs and E_2_ in follicular fluid may have regulated follicular development and ovulation.

Oxidizing and reducing reactions occur constantly in cells as part of normal aerobic functions, and the imbalance of pro-oxidants and antioxidant capacity leads to oxidative stress which is harmful to the structure, metabolism, and physiology of an organism [34]. The link between oxidative stress and reproductive performance is well established, where oxidative stress is implicated in suboptimal reproductive performance from the earliest stages of development to delivery [35]. In this study, the gilts in the SPV group had significantly higher serum concentrations of T-SOD and lower serum concentrations of MDA compared to the CON group. Feeding gilts with with sweet potato vine could improve the antioxidant capacity and abrogate the oxidative stress. Being rich in vitamins (such as VA, VC, and carotene, Table 1), sweet potato vine supplementation could provide the gilts with extra vitamins, especially VC (246 mg/d in SPV group) which is lacking in the basic diet. Earlier investigations demonstrated the role of a variety of vitamins in increasing the capability of antioxidant systems to eliminate free radicals [36,37,38]. Furthermore, the results of RT-PCR indicate that the mRNA expression in GCs conformed to the antioxidant parameter in serum, whereby GCs increased the antioxidative enzyme activities to maintain the balance of cellular oxidizing and reducing actions. These results indicate that sweet potato vine may be beneficial to particular aspects of the antioxidant system of Meishan gilts.

It is reported that the ovary is highly sensitive to oxidative stress [39]. Previous studies have indicated that a decrease in the amount of antioxidants in the follicular fluids causes deterioration of oocytes [40], and severe oxidative stress perturbs microenvironments in and around oocyte and GCs [41], thereby effectively impairing the ovarian development in pigs [42]. Moreover, gilts have been shown to sustain higher oxidative stress conditions than multiparous sows [43]. In this study, compared with the control group, gilts fed sweet potato vine had increased *SOD1* mRNA expression in the GCs and better follicular development of the gilts.

## 5. Conclusions

In conclusion, our results suggest that feeding sweet potato vine instead of part of basal diet with the same amount of energy and protein to Chinese Meishan gilts delayed the onset of puberty and improved the follicular development by regulating E_2_ and IGF-1 concentrations in follicular fluid, and *LHR* mRNA expression and T-SOD and MDA concentrations in granulosa cells.

## Figures and Tables

**Table 1:** Composition and nutrient level of basal experimental diet (as-fed basis), chemical composition of sweet potato vine for the Chinese Meishan gilts.

	Basal Experimental Diet ^1^	Sweet Potato Vine ^2^
Ingredient, %		
Corn, 7.8% CP	64.79	-
Soybean meal, 44.2% CP	14.42	
Wheat bran	18.00	
L-Lysine-HCL (75%)	0.06	
Calcium carbonate	1.00	
Dicalcium phosphate	0.23	
Salt	0.40	
Choline chloride (50%)	0.10	
Mineral premix ^3^	0.50	
Vitamin premix ^4^	0.50	
Total	100.00	
Nutrient level		
DM, %	-	10.13
DE, kcal/kg	3080.00	2837.00
CP, %	14.00	14.90
T-Lys, %	0.68	-
CF, %	3.11	15.51
NDF, %	-	21.63
ADF, %	-	19.37
ADL, %	-	3.30
Ca, %	0.53	1.61
TP, %	0.48	0.47
AP, %	0.20	-
VA, mg/kg	-	15.00
VC, mg/kg	-	123.00
VE, mg/kg	-	16.00
Carotene, mg/kg	-	42.00
Fe, mg/kg	-	11.00

DE, digestible energy; CP, crude protein; T-Lys, total lysine; CF, crude fiber; Ca, calcium; TP, total potassium; AP, available potassium; DM, dry matter; GE, gross energy; CP, crude protein; CF, crude fiber; NDF, neutral detergent fiber; ADF, acid detergent fiber; ADL, acid detergent lignin; VA, Vitamin A; VC, Vitamin C; VE, Vitamin E.; CON, gilts feed the basal diet; SPV, gilts fed basal diet with sweet potato vine.^1^ CP and Ca are analyzed values. DE is a calculated value. ^2^ DM, DE, CP, CF, NDF, ADF and ADL are analyzed values. Other data are cited from “Chinese common food nutrition”, Zou, 1991 and Zhang et al. 2003 [16,17]. VA, VC, VE and Carotene are the fresh base, others index (except DM) are the dry matter base. ^3^ Supplied per kg of mineral premix: Cu, 5.5 mg; Fe, 100 mg; I, 0.2 mg; Mn, 3.6 mg; Se, 0.3 mg; Zn, 100 mg. ^4^ Supplied per kg of vitamin premix: 0.5 mg vitamin A; 0.75 mg vitamin D3; 10 mg vitamin E; 0.5 mg vitamin K; 1 mg vitamin B1; 2.5 mg vitamin B2; 8 mg pantothenic acid; 12 mg niacin; 1.5 mg vitamin B6; 0.04 mg biotin; 0.3 mg folacin; 13 μg vitamin B12.

**Table 2:** The feeding strategy for the Chinese Meishan gilts.

	CON	SPV
Basal diets, kg/d	1.64	1.46
Sweet potato vine (fresh base), kg/d	--	2.00
DE intake, kcal/d	5051.00	5072.00
CP intake, g/d	230.00	234.00
CF intake, g/d	51.00	73.94

CON, gilts feed the basal diet; SPV, gilts fed basal diet with sweet potato vine.

**Table 3:** Primer sequences of the target and reference genes.

Gene ^1^	Primers	Sequence (5′-3′)	Accession Number	Product Size (bp)
*GPx*	Forward	GCTCGGTGTATGCCTTCTCT	NM_214201.1	103
	Reverse	AGCGACGCTACGTTCTCAAT		
*SOD1*	Forward	GAGCTGAAGGGAGAGAAGACAGT	NM_001190422.1	116
	Reverse	GCACTGGTACAGCCTTGTGTAT		
*SOD2*	Forward	CTGGACAAATCTGAGCCCTAAC	NM_214127.2	118
	Reverse	GACGGATACAGCGGTCAACT		
*FSHR*	Forward	TCACAGTCCCTCGGTTCCTT	NM_214386.1	152
	Reverse	AGCATCACAGCCTGCTCCA		
*LHR*	Forward	ATGGGGCTCTACCTGCTACTCA	NM_214449.1	255
	Reverse	GAGCCACCCTCCAAGCATAA		
*ERα*	Forward	ATGAAGTGCAAGAACGTGGTG	NM_214220.1	151
	Reverse	AATGCGATGGAGTTGAGCC		
*β-actin*	Forward	GGCCGCACCACTGGCATTGTCAT	DQ845171.1	104
	Reverse	AGGTCCAGACGCAGGATGGCG		

^1^ Gene abbreviations: *GPx*, glutathione peroxidase; *SOD1*, superoxide dismutase-1; *SOD2*, superoxide dismutase-2; *FSHR*, follicle-stimulating hormone receptor; *LHR*, luteinizing hormone receptor; *ERα*, estrogen receptor-α.

**Table 4:** Effect of sweet potato vine supplementation on estrus in Meishan gilts.

	CON	SPV	*p*-Value
Initial body weight, kg	30.30 ± 0.30	30.20 ± 0.30	0.92
Body weight at first estrus, kg	38.80 ± 1.30	42.80 ± 1.80	0.09
Age at puberty, d	135.30 ± 3.10	144.70 ± 3.90	0.08
Slaughtered body weight, kg	62.20 ± 2.20	70.20 ± 3.40	0.07
Slaughtered age, d	197.70 ± 2.30	207.40 ± 4.90	0.09
Duration of the first estrus cycle, d	21.00 ± 0.60	21.10 ± 0.40	0.89
Duration of the second estrus cycle, d	20.80 ± 0.60	20.70 ± 0.50	0.92

CON, gilts feed the basal diet; SPV, gilts fed basal diet with sweet potato vine. Values are mean ± SEM (*n* = 10).

**Table 5:** Effect of sweet potato vine supplementation on the reproductive organ development and total follicle population in Meishan gilts.

	CON	SPV	*p*-Value
Ovarian weight, g	6.00 ± 0.70 ^b^	8.10 ± 0.30 ^a^	0.01
Ovarian relative weight, g/kg	0.095 ± 0.008 ^b^	0.116 ± 0.004 ^a^	0.05
Uterus weight, g	349.60 ± 56.00 ^b^	473.80 ± 23.30 ^a^	0.04
Uterus relative weight, g/kg	5.8 ± 0.90	6.80 ± 0.70	0.35
No. Large follicles (> 5mm)	12.60 ± 1.50 ^b^	16.40 ± 0.90 ^a^	0.04
No. Medium follicles (≥ 3mm, ≤ 5mm)	4.10 ± 1.00	4.70 ± 1.20	0.76
No. Small follicles (< 3mm)	26.80 ± 5.00	30.90 ± 7.60	0.67
No. Corpora lutea	15.00 ± 4.60	25.30 ± 1.00	0.06

CON, gilts feed the basal diet; SPV, gilts fed basal diet with sweet potato vine. Values are mean ± SEM (*n* = 10). ^a,b^ Means not sharing identical superscripts in the same row are significantly different (*p* ≤ 0.05).
